# Retraction note: Down-regulation of microRNA-26a and up-regulation of microRNA-27a contributes to aggressive progression of human osteosarcoma

**DOI:** 10.1186/s13000-016-0564-5

**Published:** 2016-11-02

**Authors:** Afshin Taheriazam, Reza Bahador, Seyyed Hasan Karbasy, Seyed Mir Mansoor Moazen Jamshidi, Ali Torkaman, Emad Yahaghi, Mohammadreza Shakeri

**Affiliations:** 1 Department of Orthopedics Surgery, Tehran Medical Sciences Branch, Islamic Azad University, Tehran, Iran; 2 Department of Orthopaedic and Trauma Surgery, Birjand University of Medical Sciences, Birjand, Iran; 3 Department of Anesthesiology, Birjand University of Medical Sciences, Birjand, Iran; 4 Department of Orthopedics, Zanjan University of Medical Sciences, Zanjan, Iran; 5 Department of Orthopedics, Firoozgar Hospital, Iran University of Medical Sciences, Tehran, Iran; 6 Department of Molecular Biology, Baqiyatallah University of Medical Sciences, Tehran, Iran

## Retraction

The Editor-in-Chief and Publisher have retracted this article [1] because the scientific integrity of the content cannot be guaranteed. An investigation by the Publisher found it to be one of a group of articles we have identified as showing evidence suggestive of attempts to subvert the peer review and publication system to inappropriately obtain or allocate authorship. This article showed evidence of plagiarism (most notably from the articles cited [2–4]) and authorship manipulation.
